# *Panicum Mosaic Virus* and Its Satellites Acquire RNA Modifications Associated with Host-Mediated Antiviral Degradation

**DOI:** 10.1128/mBio.01900-19

**Published:** 2019-08-27

**Authors:** Jesse D. Pyle, Kranthi K. Mandadi, Karen-Beth G. Scholthof

**Affiliations:** a Department of Plant Pathology & Microbiology, Texas A&M University, College Station, Texas, USA; b Texas A&M AgriLife Research & Extension Center, Texas A&M University System, Weslaco, Texas, USA; University of Nebraska–Lincoln; University of Texas at San Antonio; University of Missouri

**Keywords:** *Panicum mosaic virus*, polyadenylation, positive-sense RNA virus, RNA degradation, satellite virus

## Abstract

Positive-sense RNA viruses in the *Tombusviridae* family have genomes lacking a 5′ cap structure and prototypical 3′ polyadenylation sequence. Instead, these viruses utilize an extensive network of intramolecular RNA-RNA interactions to direct viral replication and gene expression. Here we demonstrate that the genomic RNAs of *Panicum mosaic virus* (PMV) and its satellites undergo sequence modifications at their 3′ ends upon infection of host cells. Changes to the viral and subviral genomes arise *de novo* within *Brachypodium distachyon* (herein called *Brachypodium*) and proso millet, two alternative hosts of PMV, and exist in the infections of a native host, St. Augustinegrass. These modifications are defined by polyadenylation [poly(A)] events and significant truncations of the helper virus 3′ untranslated region–a region containing satellite RNA recombination motifs and conserved viral translational enhancer elements. The genomes of PMV and its satellite virus (SPMV) were reconstructed from multiple poly(A)-selected *Brachypodium* transcriptome data sets. Moreover, the polyadenylated forms of PMV and SPMV RNAs copurify with their respective mature icosahedral virions. The changes to viral and subviral genomes upon infection are discussed in the context of a previously understudied poly(A)-mediated antiviral RNA degradation pathway and the potential impact on virus evolution.

## Abstract (precis)

The genomes of positive-sense RNA viruses have an intrinsic capacity to serve directly as mRNAs upon viral entry into a host cell. These RNAs often lack a 5′ cap structure and 3′ polyadenylation sequence, requiring unconventional strategies for cap-independent translation and subversion of the cellular RNA degradation machinery. For tombusviruses, critical translational regulatory elements are encoded within the 3′ untranslated region of the viral genomes. Here we describe RNA modifications occurring within the genomes of *Panicum mosaic virus* (PMV), a prototypical tombusvirus, and its satellite agents (i.e., satellite virus and noncoding satellite RNAs), all of which depend on the PMV-encoded RNA polymerase for replication. The atypical RNAs are defined by terminal polyadenylation and truncation events within the 3′ untranslated region of the PMV genome. These modifications are reminiscent of host-mediated RNA degradation strategies and likely represent a previously underappreciated defense mechanism against invasive nucleic acids.

## INTRODUCTION

Viruses of eukaryotes face constant pressure to evade the intrinsic defenses of the host cell, including nucleic acid sensors, molecular turnover machinery, and induced immune factors. These selective forces favor viral populations that are adept at making “nonself” look like “self” during infection. Positive-sense RNA viruses have an arguable advantage in this regard, primarily due to the coding orientation of their infectious genomic RNAs and their ability to directly engage the host translational machinery.

The *Tombusviridae* is a large family of positive-sense RNA viruses with many species capable of infecting evolutionarily diverse host plants and replicating in numerous cell types (1, 2). Tombusviruses have relatively small genomes (∼4-kb single-stranded RNA) and encode a minimal assortment of viral proteins (∼4 to 7). These features make tombusviruses ideal models for the study of positive-sense RNA virus replication and gene expression and the fundamental cellular factors exploited during these processes. Tombusvirus genomes must serve directly as mRNAs to initiate infection, yet they do not contain prototypical eukaryotic 5′ m^7^GpppN cap structures or 3′ polyadenylation [poly(A)] sequences (3–5). To overcome this issue, tombusviruses encode a diverse suite of structured RNAs for cap-independent translation, most of which are located in the 3′ untranslated region (UTR) of the viral genomes (6–14). The absence of a protective poly(A) tail and additional mRNA binding proteins renders these elements susceptible to host RNase activity in the cytoplasm (15–18).

**FIG 1:**
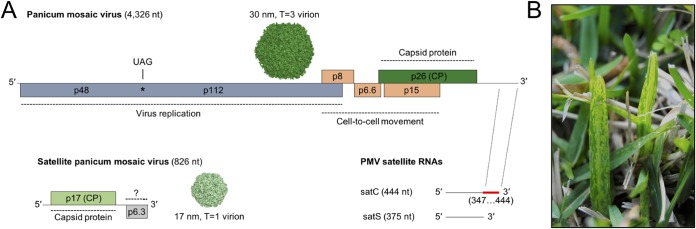
The tripartite panicovirus pathosystem. (A) Genome organization of *Panicum mosaic virus* (top), satellite panicum mosaic virus (bottom left), and the PMV satellite RNAs (bottom right). Open reading frames and protein names are indicated within the colored boxes. The position of the UAG amber stop codon in the PMV genome is indicated by an asterisk. The SPMV putative ORF2 of unknown function is indicated by a question mark. The region of shared sequence similarity between the 3′ ends of PMV and satC (nt positions 347 to 444) RNAs is shown on the satC genome in red. Surface representations of the PMV (PDB 4V99) and SPMV (PDB 5CW0) virion biological assemblies are shown in forest green (top) and pale green (bottom), respectively. (B) Diseased St. Augustinegrass (*Stenotaphrum secundatum*) turfgrass with typical symptoms of St. Augustine decline caused by PMV and its satellite agents.

*Panicum mosaic virus* (PMV; genus *Panicovirus*) is a prototypical tombusvirus with a 4.3-kb single-stranded RNA genome encapsidated within ∼30-nm icosahedral virions (Fig. 1A). The structure and function of the PMV 3′ translation enhancer element has been studied extensively, and related elements are present within numerous tombusvirus genomes (11, 12, 14, 19–21). The PMV genome encodes only six viral proteins: two replication-associated proteins (p48 and the p112 translational read-through product), three cell-to-cell movement proteins (p8, p6.6, and p15), and a 26-kDa capsid protein (Fig. 1A) (22–24). In the 1970s, PMV was found to be the associated causal agent of St. Augustine decline disease of St. Augustinegrass (Stenotaphrum secundatum) (Fig. 1B) and has recently reemerged as the predominant viral pathogen of bioenergy switchgrass (Panicum virgatum) (25–27). Within an infected cell, PMV frequently supports the replication of distinct subviral agents, including a satellite virus (SPMV) with a 0.8-kb genome and 0.3- to 0.5-kb satellite RNA populations (Fig. 1A) (25, 26, 28–31). These satellites have a range of host-specific effects on the PMV-induced disease phenotype, including symptom exacerbation (SPMV coinfection) and attenuation (satellite RNA coinfection) (28, 29, 32, 33). During mechanical transmission to a new host, the satellite RNAs are preferentially packaged by the 17-kDa capsid protein of SPMV, thus promoting maintenance of the tripartite pathosystem in nature (Fig. 1) (30, 34).

The molecular interactions of disease that underlie the PMV pathosystem are studied extensively using alternative hosts in the laboratory, notably Brachypodium distachyon (herein called *Brachypodium*), green foxtail millet (Setaria viridis), and proso millet (Panicum miliaceum) (28, 29, 32, 35–38). Previously we characterized the *Brachypodium* transcriptomic changes to infections by diverse positive-sense RNA viruses and specifically the disease synergism induced by coinfection of PMV and SPMV (32, 35). PMV and SPMV alter the regulation of defense hormone signaling pathways (i.e., salicylic acid, jasmonic acid, ethylene) and immune-related transcription factors (e.g., WRKY), as well as global changes to host mRNA splicing events (32, 35, 36, 39–41).

Here, we present the serendipitous discovery that the genomes of PMV and its satellite agents undergo 3′-end modifications within the infected host cell. Viral and subviral RNAs were detected in poly(A)-selected *Brachypodium* transcriptome samples, and full genomes were reassembled from poly(A)-selected data sets deposited in the National Center for Biotechnology Information (NCBI) Sequence Read Archive (SRA). For PMV, cloned cDNAs from infected *Brachypodium* tissues contained major 3′-end truncations encompassing the entire UTR, with added heterogeneous bases and noncanonical poly(A) tails. Polyadenylated PMV, SPMV, and satellite RNA genomes were also isolated from naturally infected St. Augustinegrass tissues, suggesting that this phenomenon is not an artifact of laboratory infections. We also find polyadenylated PMV and SPMV RNAs associated with purified virions from transcript-inoculated proso millet hosts, demonstrating that these altered genomes retain packaging signals for effective transmission. The modified RNAs share hallmarks with products of host-mediated RNA decay pathways and may be the result of previously underappreciated host antiviral responses to infection.

## RESULTS

### Viral and subviral RNAs are modified upon transcript-mediated inoculation.

**FIG 2:**
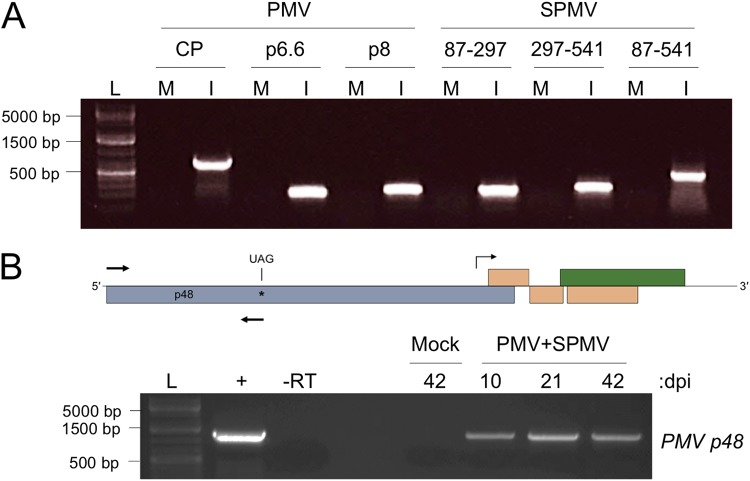
PMV and SPMV RNAs are polyadenylated *in vivo* during infection of *Brachypodium*. (A) Amplification of PMV and SPMV cDNAs from transcript-inoculated *Brachypodium*. Primers corresponding to the three PMV ORFs (CP, p6.6, and p8) and the three regions of the SPMV genome (positions 87 to 297, 297 to 541, and 87 to 541) were used to amplify cDNA from mRNA-enriched transcripts purified from infected plant tissues. Lanes M, reaction mixtures containing cDNA from mock-infected plant tissues; lanes I, reaction mixtures containing cDNA from PMV-SPMV-infected plant tissues. (B) PMV RNA containing the p48 ORF is polyadenylated during infection of *Brachypodium*. (Top) A genome schematic for PMV, indicating the forward and reverse primer position for amplification of the p48 ORF (arrows). The subgenomic RNA promoter region is indicated by a bent arrow. (Bottom) Amplification of the PMV p48 ORF from mock- and PMV-SPMV-infected *Brachypodium* cDNA at 10, 21, or 42 days postinoculation (dpi). Lane +, a PCR mixture containing the PMV infectious cDNA plasmid as a control for amplification; lane -RT, a reaction mixture with PMV-SPMV cDNA at 42 dpi where no reverse transcriptase enzyme was included for the cDNA synthesis reaction. Lanes L for panels A and B contain a DNA molecular weight marker.

Previously, we utilized *Brachypodium distachyon* as an alternate host to study the misregulated transcriptome resulting from PMV and PMV-plus-SPMV (PMV-SPMV) infections (32, 36). These plants were mechanically inoculated with transcripts synthesized *in vitro* from linearized PMV and SPMV infectious cDNA clones. Total RNA was purified from mock-, PMV-, and PMV-SPMV-infected *Brachypodium* tissues at ∼10 to 14 days postinoculation and subjected to microarray analysis, as well as transcriptome sequencing (RNA-seq), using mRNA-enriched libraries (32, 36). In order to validate the transcriptome changes by reverse transcription (RT)-PCR, total RNA was converted to cDNA using an oligo(dT) primer, followed by PCR of selected genes (32, 36). Inadvertently, we used the oligo(dT)-primed cDNA to detect PMV and SPMV in the inoculated plants. Because PMV and SPMV are presumably nonpolyadenylated, we were surprised to find that multiple PMV and SPMV gene-specific products were readily amplified using the oligo(dT)-primed cDNA in the RT-PCR assay (Fig. 2A). This serendipitous result suggested that the oligo(dT) primer possibly also hybridized to stretches of adenine bases present within the PMV and SPMV RNAs, despite the absence of such sequences in the original infectious clones.

PMV expresses its capsid and movement proteins using a single subgenomic RNA synthesized by the viral RNA-dependent RNA polymerase (RdRP) (22, 23). It is possible that our initial RT-PCR detection of PMV polyadenylated viral RNAs corresponding to CP, p6.6, and p8 open reading frames (ORFs) could reflect only polyadenylation of the subgenomic RNA (Fig. 2A). Primers for amplification of p48, the only ORF product entirely encoded by genomic RNA, was used for the semiquantitative RT-PCR (23, 24). The results revealed that p48 could be readily amplified from the oligo(dT)-primed cDNA corresponding to PMV-infected *Brachypodium* samples collected at 10, 21, and 42 days postinfection (Fig. 2B).

**FIG 3:**
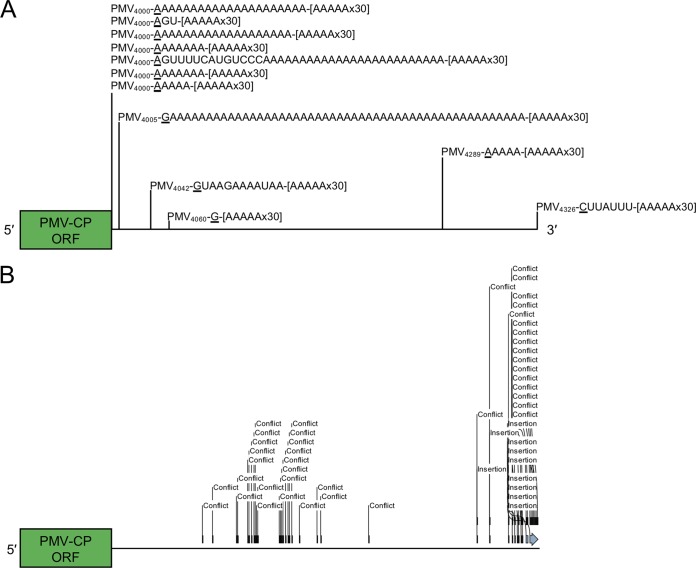
The PMV 3′ UTR is notably edited in *Brachypodium*. (A) mRNA-enriched PMV RNAs contain major truncations and A/U-rich sequences in their 3′ UTRs. The 3′ end of the PMV capsid protein (CP) ORF is shown in green, followed by a horizontal line representing the 3′ UTR. Relative positions of the 3′ termini from 12 mRNA-enriched clones are indicated with vertical lines. The position of the truncated termini within the PMV genome is indicated by subscript numbers. Terminal residues corresponding to the truncation point in the wild-type genome are underlined. Nonviral sequences are shown to the right of the underlined residue, followed by an indicator for the RT-PCR oligo(dT) reverse primer ([AAAAAx30]). (B) Insertion and substitution events within the PMV 3′ UTR identified from short reads in the SRA. The scale of the UTR is the same as described for panel A. “Conflict” indicates substitution or deletion events; “insertion” indicates base insertion events.

To further validate and determine the sequence(s) of these apparent poly(A) features within the viral genomes, we sequenced the 3′ ends of PMV products amplified using the oligo(dT)-primed cDNA. Sanger sequencing analyses of 12 individual clones revealed that most products contained several A- or U-rich stretches of added bases (∼30 bases or more) downstream of the viral sequences, with few containing heterogeneous sequences of unknown origin. We also noted several truncations at approximate nucleotide position 4000 within the 3′ UTR of the viral RNA, followed by stretches of A/U-rich bases (Fig. 3A). A single isolated clone contained an A/U-rich region added precisely at the 3′ terminus of the PMV genome at nt 4326, suggesting that truncation of the viral RNA is not necessarily a prerequisite for nonviral sequence addition (Fig. 3A).

Together, these findings demonstrate that PMV and SPMV RNAs are polyadenylated in *Brachypodium* and support the notion that tombusvirus RNAs can be processed for *de novo* polyadenylation and 3′ UTR truncation/editing events during infection of host plants.

### Assembly of viral and subviral genomes from poly(A)-selected transcriptome data sets.

After completion of the PMV-*Brachypodium* RNA-seq study (36), using the mRNA-enriched libraries, we deposited the raw reads in the Sequence Read Archive (SRA) at the National Center for Biotechnology Information (NCBI). Our preliminary observations demonstrated that poly(A) forms of the PMV and SPMV genomes are present within these transcriptomes (Fig. 2 and 3A). To probe the *Brachypodium* transcriptome further, we leveraged our RNA-seq data set in order to data-mine and identify hits from the poly(A)-selected data set corresponding to the PMV and SPMV viral and subviral genomes. Using the reference genomes for PMV (accession no. NC_002598.1) and SPMV (accession no. NC_003847.1) as search queries, we identified abundant viral and subviral raw reads from the PMV-infected (accession no. SRX747740) and PMV-SPMV-infected (accession no. SRX747746) *Brachypodium* SRA data sets. The SRA search parameters and results are summarized in Table S1 in the supplemental material.

10.1128/mBio.01900-19.3 TABLE S1 Summary of Sequence Read Archive (SRA) mining search parameters and results. Download Table S1, DOCX file, 0.02 MB. Copyright © 2019 Pyle et al. 2019 Pyle et al. This content is distributed under the terms of the Creative Commons Attribution 4.0 International license.

From the PMV-SPMV-infected *Brachypodium* transcriptome, we identified ∼20,000 reads with strong sequence similarity to the PMV reference genome (Table S1). These reads were *de novo* assembled into a single contiguous consensus sequence (contig). This contig was 4,397 nt in length, 71 bases longer than the PMV reference genome, with an extension of 23 bases from the 5′ end of the viral genome. This 5′ extension is identical to the sequence from nt positions 125 to 147 in the original PMV clone and possibly reflects an artifact of the contig assembly process. The wild-type 3′ end was present in the assembled contig, followed by 26 adenine residues downstream of the 5′-ACCAGGCCC-3′ terminal motif (30). A 22-base sequencing adapter was also identified in the PMV contig, downstream of the poly(A) sequence. A blastn search using the *de novo* assembled contig revealed a very high degree of sequence similarity with the PMV reference genome, with only three base substitution events, excluding the additional 5′- and 3′-end extensions (C848A, A1799C, and U3752C). The C848A and U3752C substitutions result in synonymous leucine-to-leucine mutations in the p48 and p26 proteins, respectively. The A1799C substitution results in a nonsynonymous threonine-to-proline mutation at amino acid position 591 in p112. The origin of this point mutation in the major PMV replication-associated protein is unclear, but we hypothesize that it could be an artifact of RNA sequencing and/or base calling algorithms. Alternatively, it could be a valid mutation in the genome that occurred during PMV replication in plants and whose functional consequences remain to be determined. Nevertheless, the blastn search yielded significant hits to related panicoviruses (e.g., *Thin paspalum asymptomatic virus*, *Cocksfoot mild mosaic virus*, *Bermuda grass latent virus*) and the 3′ ends of the PMV satellite RNAs, which originate from recombination events with the helper virus genome during infection (28, 30), all of which further underscore the notion that PMV sequences are adenylated/modified *de novo*.

A similar approach was taken to reveal polyadenylation of SPMV, where we probed the PMV-SPMV-infected *Brachypodium* transcriptome to retrieve reads corresponding to the SPMV reference genome. Although fewer reads were recovered in this data set (763 reads) than in the PMV data set, a single contig of 836 nt in length corresponding to SPMV was reconstructed using these reads for the *de novo* assembly. In a manner similar to that of PMV, the SPMV contig was 10 bases longer than the SPMV reference sequence and contained many mutations throughout the genome, including multiple deletion and substitution events (Fig. S1). The SPMV contig also had an extension of 16 bases at the 5′ end (5′-CCGGGCTMCYGSSRCA-3′), which has no apparent similarity to sequences in NCBI. Unlike the PMV contig, the SPMV contig was missing all 5 bases of its short 3′ UTR downstream of the stop codon from the ORF for a small ∼6.3-kDa putative SPMV-encoded protein (Fig. 1A and Fig. S1). Moreover, the SPMV contig did not contain any stretch of adenine residues at the 3′ end. These differences may be explained by the relatively low number of reads identified and used for generation of the SPMV contig relative to the reads identified for PMV. A blastn probe using the SPMV contig revealed expected hits, including the previously characterized SPMV defective interfering RNAs (42, 43).

10.1128/mBio.01900-19.1 FIG S1 Sequence alignment of the SPMV reference genome (SPMV_ref, NC_003847.1) and the assembled SPMV contig from an mRNA-enriched *Brachypodium* transcriptome dataset (SPMV_contig). The SPMV p17 capsid protein ORF and p6.3 putative ORF are highlighted in blue and yellow, respectively. The 3′ terminus of the reference genome and nonviral sequences at the 5′ end of the assembled contig are underlined. Mismatches between the sequences are indicated by the absence of a vertical line. Dashes indicate sequence gaps. Sequence limits are indicated on either side of the alignment panel. Lowercase “G” bases in grey for SPMV_ref represent a region of the query that was automatically filtered for low sequence complexity according to the NCBI blastn suite parameters. Download FIG S1, JPG file, 1.4 MB. Copyright © 2019 Pyle et al. 2019 Pyle et al. This content is distributed under the terms of the Creative Commons Attribution 4.0 International license.

Since the PMV 3′ UTR showed various truncations, editing, and poly(A) events *in vivo* (Fig. 3A), we next asked whether mutations could be observed in the individual RNA-seq reads, mutations that would otherwise be masked during consensus contig assembly. While most of the PMV RNAs in our Sanger sequencing experiments were defined by significant truncations within the 3′ UTR with stretches of poly(A) tails (Fig. 3A), cDNA fragmentation during RNA-seq library preparation and the stringent mapping/assembly parameters preclude identification of such extended poly(A) sequences from the SRA data set. However, the fact that we were able to assemble full-length PMV genomes from poly(A)-selected libraries, and further sequence analyses of short reads corresponding to the ∼300 bases of the PMV 3′ UTR-identified abundant base substitution and insertion events in the RNA-seq data set, lends support to the Sanger sequencing that the 3′ terminus is edited (Fig. 3B and Fig. S2). Most of these mutations clustered proximal to the 3′ terminus or ∼150 bases upstream within the PMV cap-independent translational enhancer element of the 3′ UTR (Fig. 3B and Fig. S2) (11). These results suggest that although PMV quasispecies in *Brachypodium* is predominantly representative of the wild-type viral genome, diverse RNA species which acquire various mutations, including *de novo* polyadenylation during infection, do exist.

10.1128/mBio.01900-19.2 FIG S2 Detailed analysis of mutation sites in the PMV 3′ UTR identified from the *Brachypodium* transcriptome data set. Individual reads from the PMV-SPMV-infected *Brachypodium* data set were compared against the 3′ UTR of the PMV reference genome (NC_002598.1). The 3′ end of the PMV capsid protein ORF is shown in green. The region corresponding to the 3′ cap-independent translation enhancer is underlined in blue. Base mismatches or insertions are indicated as “Conflict” or “Insertion,” respectively. Residue labeling follows the standard expanded nucleic acid letter code to indicate the nature of each mutation. Download FIG S2, JPG file, 0.5 MB. Copyright © 2019 Pyle et al. 2019 Pyle et al. This content is distributed under the terms of the Creative Commons Attribution 4.0 International license.

### Viral and satellite poly(A) RNAs persist during natural infections.

**FIG 4:**
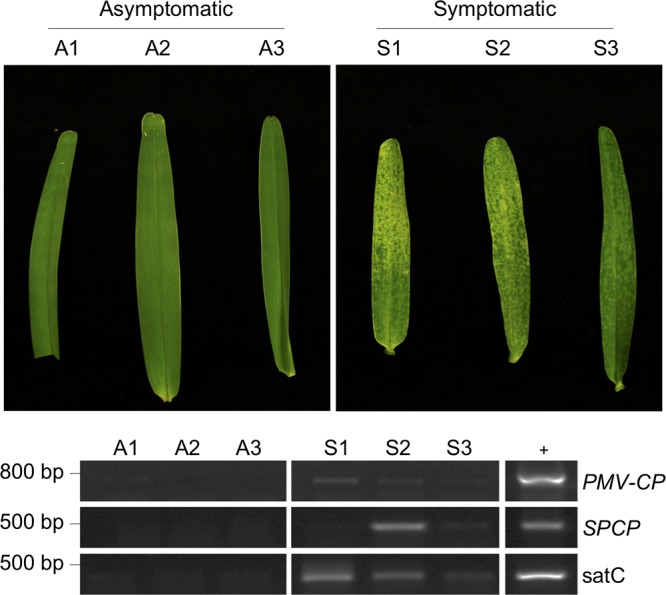
PMV, SPMV, and satC RNAs are polyadenylated during natural infections of St. Augustinegrass. (Top) Three asymptomatic (A1 to A3) and symptomatic (S1 to S3) St. Augustinegrass leaf samples were collected from the Texas A&M University campus. Symptomatic samples were selected based on the typical chlorotic mottling associated with PMV infection and St. Augustine decline disease. (Bottom) Semiquanititative RT-PCR detection of PMV, SPMV, and satC cDNA from mRNA-enriched RNA purified from the six St. Augustinegrass tissue samples. Sample cDNAs were synthesized using oligo(dT) primers for reverse transcription. Primers for the PMV capsid protein ORF (PMV-CP*)*, the SPMV capsid protein ORF (SPCP), and satC were used for amplification of PMV, SPMV, and satC cDNAs, respectively (see Table S2 in the supplemental material). Lane +, PCR mixtures containing the PMV, SPMV, or satC infectious cDNAs as positive controls for amplification.

We next inquired whether the modifications to the viral and subviral genomes was limited to only infections in *Brachypodium* and/or was a result of mechanical inoculation of T7-synthesized RNAs. To address this, we analyzed leaf tissues of naturally infected St. Augustinegrass (*Stenotaphrum secundatum*), a C_4_ warm-season turfgrass that is phylogenetically divergent from *Brachypodium*, a temperate C_3_ grass. St. Augustinegrass is a very prevalent host for PMV and SPMV in the Gulf Coast region of the United States (25, 26). The natural populations of St. Augustinegrass have been independently verified as hosts for infections by PMV and its satellite agents for at least 2 decades (25, 26, 28). Samples were collected from PMV-SPMV-infected St. Augustinegrass on the campus of Texas A&M University, College Station, TX. RT-PCR with oligo(dT)-primed cDNAs revealed that tissues displaying the characteristic symptoms of St. Augustine decline amplified positive for PMV and SPMV (Fig. 4). Interestingly, the St. Augustinegrass samples also contained the chimeric satellite RNAs of PMV, satC RNAs, which were also readily amplified, suggesting that they could be polyadenylated (28, 30). Three asymptomatic samples did not test positive for any of the three agents, confirming again the association of PMV and its satellites with the disease symptoms of St. Augustinegrass (Fig. 4).

10.1128/mBio.01900-19.4 TABLE S2 Sequences of oligonucleotides used in the current study. Forward and reverse primers for each set are indicated with “-F” and “-R” trailing the primer name, respectively. Download Table S2, DOCX file, 0.01 MB. Copyright © 2019 Pyle et al. 2019 Pyle et al. This content is distributed under the terms of the Creative Commons Attribution 4.0 International license.

### Poly(A) RNAs are associated with purified PMV and SPMV virions.

**FIG 5:**
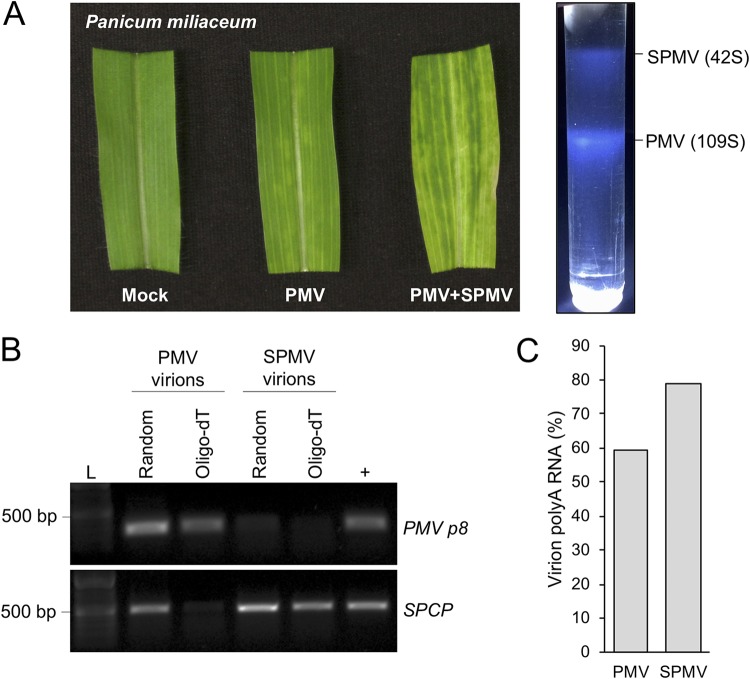
The polyadenylated RNAs of PMV and SPMV are associated with purified virions from infected proso millet (*Panicum miliaceum*) hosts. (A) Purification of PMV and SPMV virions from infected proso millet. (Left panel) A comparison of symptoms among mock-, PMV-, and PMV-SPMV-infected proso millet. (Right panel) Typical sucrose density gradient showing the sedimentation patterns of purified SPMV (42S) and PMV (109S) virions. (B) Semiquantitative RT-PCR analyses of PMV and SPMV RNAs from purified virions. Purified virion RNAs were subjected to cDNA synthesis primed with random hexamers or oligo(dT) primers. PMV p8 and SPCP cDNA ORFs were amplified for detection of the PMV and SPMV RNAs, respectively. Lane L, DNA molecular weight marker; lane +, products from reaction mixtures containing PMV or SPMV cDNA plasmid templates as positive controls for the PCRs. (C) Relative percentage of poly(A) RNAs packaged within PMV and SPMV virions. Quantification was performed by comparing the relative abundance ratios of amplified cDNA products from oligo(dT)- and random hexamer-primed RT reactions for each of the purified virions. Products of cDNA amplification were quantified using ImageJ.

The infectious RNAs of PMV and its satellites are transmitted to susceptible hosts within small icosahedral virions (29–31, 34, 44–46). The PMV and SPMV genomes encode separate capsid proteins to form T=3 and T=1 virions, respectively (Fig. 1A). PMV satellite RNAs can be packaged by either of these structures but are preferentially encapsidated by the SPMV capsid protein, thus maintaining the tripartite pathosystem (25, 30, 34). Packaging of the poly(A) RNAs, most of which are presumably defective templates for replication and recognition by cellular translation machinery, would have implications for effective transmission and initiation of subsequent infections and the population-level effect of a viral quasispecies bottleneck. To address this possibility, we analyzed the RNA contents of purified PMV and SPMV virions from transcript-inoculated proso millet (*Panicum miliaceum*), another well-established alternate host of PMV and SPMV (11, 22–24, 42, 47, 48). Virions were purified by sequential ultracentrifugation and sucrose density gradient sedimentation (Fig. 5A), and the virion-associated RNAs were subjected to semiquantitative RT-PCR analyses.

Poly(A) RNAs were detected in purified PMV and SPMV virions via amplification of oligo(dT)-primed cDNAs (Fig. 5B). In comparison to the corresponding abundances of random hexamer-primed cDNAs, the oligo(dT)-primed cDNA fractions were approximately 60% and 80% of virion-associated cDNAs for PMV and SPMV, respectively (Fig. 5C). A fraction of the SPMV virion-associated RNA also copurified with the PMV virions (Fig. 5B, second lane from left), suggesting packaging of the SPMV genome by the PMV capsid protein, an observation supported by previously determined RNA binding properties of the PMV capsid protein (34). In summary, these results demonstrate that the RNA genomes of PMV and SPMV, and likely the noncoding PMV satellite RNAs, are polyadenylated within multiple native and alternative hosts during infection following either mechanical inoculations or natural infections.

## DISCUSSION

The principle finding of this study is that the RNAs of *Panicum mosaic virus* and its two distinct satellite agents are modified in a previously unknown way, challenging our understanding of tombusvirus replication events and viral population dynamics within the infected hosts. For PMV, these alterations are defined by *de novo* polyadenylation and extensive editing (single nucleotide polymorphisms [SNPs], indels) events within the 3′ UTR. Furthermore, we found that the modifications occur in both laboratory and natural infections of multiple hosts (native and alternative), underscoring its relevance beyond a single host-virus pathosystem.

### Polyadenylation of viral RNAs: pro- or antiviral?

Our initial hypothesis for the polyadenylation involved virus-mediated acquisition of 3′ poly(A) tails in order to self-protect from host RNA degradation or surveillance mechanisms, an inherently proviral mechanism. This “tail-snatching” strategy was hypothesized to be analogous to cap-snatching, a process mediated by the RdRPs of segmented negative-sense RNA viruses for acquisition of capped primers for synthesis of viral mRNAs (49–51). Cap-snatching involves a virus-encoded endonuclease within the RdRP, an enzymatic activity that has not been associated with any protein encoded by the PMV genome or any known tombusvirus protein. However, the genomes of PMV and SPMV are prone to inter- and intra-RNA recombination events for the generation of chimeric helper-satellite RNAs (28, 30) or defective interfering RNAs of SPMV (42, 43). Similar recombination events involving virus genomes and host mRNAs may occur to generate the polyadenylated viral and subviral RNAs detected in this study.

Currently, it remains unclear whether the modified viral RNAs enhance or attenuate PMV pathogenicity. Paradoxically, PMV is frequently associated with both SPMV and the satellite RNAs (25–28, 30), which have opposing effects on the replication of the helper virus (28, 29, 32). PMV persists for years within an infected host, suggesting a fine-tuned balance between virus replication and host defenses (27). Assuming that many of the modified PMV RNAs are defective for translation, due to loss of enhancer elements in the 3′ UTR and less initiation factor recruitment and RNA sequestration within translating polysome complexes (6, 7, 10–12, 14, 19), one might predict priming of the intrinsic host defense responses (e.g., RNA interference [RNAi], nonsense-mediated RNA decay, nucleic acid sensors) relative to a homogeneous viral RNA population with intact replication/translation signals. Moreover, poly(A) RNAs packaged within virions may serve as a quasispecies buffer in the transmission bottleneck (52), competing with infectious full-length genomes entering new host cells and limiting the genetic pool from which more-fit viral genotypes could emerge. On the other hand, the selective retention of nonpolyadenylated SPMV genomes within PMV virions (Fig. 5B) may reflect an unidentified packaging signal required in the 3′ end of the SPMV RNA, implicating a proviral strategy for retaining functional genomes. Additionally, our small-scale sequencing identified a single PMV RNA with a new AUG start codon introduced within the heterogeneous bases of unknown origin (Fig. 3A). Despite the absence of a canonical in-frame stop codon in this clone, this observation suggests that, over time, these RNA editing mechanisms may introduce novel genetic material with coding capacity into the viral quasispecies, a potential proviral evolutionary strategy. Future studies implementing high-fidelity deep-sequencing methods such as circular RNA sequencing (CirSeq) are necessary to address the consequences of *de novo* polyadenylated RNAs and low-frequency viral genome variants on quasispecies population dynamics (53). The abundance of modified invasive RNAs could represent additional regulatory measures for viral RNA accumulation, either by maintaining a basal defensive activation of host RNA interference and/or RNA degradation machinery or by limiting the pool of infectious viral and subviral RNAs transmitted to new hosts.

### Origins of the nonviral RNA sequences.

The source of nonviral RNA sequences within the PMV genome remains a mystery; however, the possible origins are not endless. One possibility is via the activity of the PMV RdRP, which is directly responsible for amplification of both helper and satellite genomes. PMV expresses its four movement- and assembly-associated proteins (i.e., p8, p6.6, p15, and p26) by using a single subgenomic RNA originating from a prematurely terminated minus-sense replication intermediate (5, 22, 23). Since most of the poly(A) clones contained stretches of nonviral adenine residues at the immediate 3′ terminus of the p26 ORF (Fig. 3A, nt position 4000), it is possible that the PMV RdRP has an intrinsic and previously unrecognized poly(A) stuttering mechanism similar to that of other viral RNA polymerases (54–56). However, previous observations from PMV-infected protoplasts suggest that the PMV RdRP synthesizes a single subgenomic RNA (1,475 nt) encompassing the full PMV 3′ UTR, leaving poly(A) stuttering as an unlikely possibility (23). Alternatively, since many viral RdRPs also possess intrinsic terminal transferase activity (57–59), including the polymerase of a distantly related tombusvirus, *Turnip crinkle virus* (60, 61), this possibility may exist for the RdRP of PMV. Given the long poly(A) tails identified in our sequencing experiments (Fig. 3A), the processive terminal transferase activity of PMV RdRp would also require some degree of substrate selectivity for ATP nucleotides for incorporation. Further studies on the enzymatic activity of purified PMV RdRPs and of related tombusviruses are required to address these functions and the subsequent fate of the edited RNAs, notably whether these transcripts are aberrant or represent viral RdRP-synthesized mRNAs with poly(A) tails for enhanced translation.

A second favorable model is the possibility that nonviral sequences are added by a host factor. In addition to the nuclear mRNA polyadenylation machinery, mitochondrion- and chloroplast-localized poly(A) polymerases (PAPs) and polynucleotide phosphorylases (PNPases) are associated with the addition of poly(A) and A-rich tails to the 3′ ends of cellular RNAs (62–66). These modified RNAs are intermediates in an ancient poly(A)-mediated RNA decay pathway from bacteria and archaea co-opted by eukaryotic cells (65–70). In addition to their roles in host RNA turnover, PAP- and PNPase-related enzymes could be repurposed for targeting viral RNAs for degradation during infection (71–73). Such *de novo* added A/U-rich sequences were indeed identified in the genomes of diverse plant and animal RNA viruses that are known to lack genomic poly(A) tails, suggesting that this mechanism is conserved among several eukaryotic hosts (72–79). The ancient poly(A)-mediated RNA decay pathway consists of a three-step mechanism involving (i) 3′-to-5′ RNA nuclease degradation, (ii) poly(A) tail addition, and (iii) poly(A)-mediated RNA degradation (65). Evidence supporting these three stages can be found from our sequencing data of the PMV 3′ UTR, where modified viral RNAs are distinguished by significant truncations, deletions, and substitutions and polyadenylation signatures (Fig. 3A and B). Recent insights into RNA decay as an antiviral strategy have demonstrated that viruses with RNA genomes are targeted by the cellular immune system for 3′ uridylation and subsequent degradation (80, 81). Cytoplasmic terminal uridylyltransferases are widely conserved among eukaryotes for functional roles in mRNA turnover, a function that could be repurposed for defense against invading RNA viruses (81). The methodology employed in the current study precludes identification of poly(U) tails present within the genomes of PMV, SPMV, or the satellite RNAs; however, our sequencing efforts did reveal nonviral U-rich sequences in the PMV genome (Fig. 3A). Nevertheless, the role of host PAPs and PNPases in the control of viral and subviral RNAs during infection requires further investigation.

A third possibility involves trafficking of the PMV, SPMV, and satellite RNAs into the host nucleus for polyadenylation. The capsid proteins of PMV and SPMV localize to the nucleus and nucleolus/Cajal-like bodies, respectively (34, 82). The purpose of this subcellular localization for capsid proteins of viruses that replicate and assemble in the cytoplasm remains a mystery. Moreover, the capsid proteins bind their respective genomic RNAs and the satellite RNAs, suggesting that their nuclear localization may translocate these RNAs to the ideal cellular compartment for polyadenylation by Pol II-associated host factors. However, this does not explain the truncations, mutations, or heterogeneous nonviral sequences identified in this study. Alternatively, a recently described family of monocot-specific PAPs, lacking nuclear localization domains, could modify the viral and subviral RNAs at the replication sites in the cytoplasm (83).

In summary, we have defined previously underappreciated *in vivo* modifications to the RNAs of PMV and its satellites. The mutation and deletion signatures within the PMV 3′ UTR implicate a host poly(A)-mediated RNA degradation strategy with antiviral potential. We hypothesize that this phenomenon is not unique to tombusviruses and may represent a host-directed RNA degradation strategy with antiviral implications. Similar observations have been sporadically reported in the literature for additional RNA viruses from diverse eukaryotic hosts, indicating that this phenomenon extends beyond the plant immune response against invasive nucleic acids. This RNA modification strategy deserves further attention in the broad context of known antiviral host responses and viral evasion mechanisms.

## MATERIALS AND METHODS

### Plant growth conditions and sampling.

*Brachypodium distachyon* (accession Bd21-3) and proso millet (*Panicum miliaceum* cv. Sunup) were grown under previously described conditions (28). Seeds were cold stratified in the dark at 4°C for 1 week to facilitate uniform germination rates. After cold stratification, the seeds were moved into growth chambers with diurnal conditions of 14 h of daylight (∼250 to 300 μmol/m^2^/s) and 10 h of darkness at 21°C and 18°C, respectively. The St. Augustinegrass (*Stenotaphrum secundatum*) leaf samples were collected from a 10-m^2^ lawn area on the Texas A&M University campus (30.615481°N, 96.338344°W), as reported previously (28), flash frozen in liquid nitrogen, and stored at –80°C until total RNA extraction.

### Synthesis of viral and subviral genomic RNAs and mechanical inoculations.

Transcripts for PMV and SPMV were prepared as described previously (23, 28, 84). Linearized cDNA plasmid clones of PMV (pPMV85) and SPMV (pSPMV1) were used for synthesis of genomic RNA transcripts by T7 RNA polymerase (NEB). PMV and PMV-SPMV inoculum was prepared by mixing the viral and subviral RNAs in a 1:1 ratio (vol/vol) with an equal volume of RNA inoculation buffer (50 mM KH_2_PO_4_, 50 mM glycine [pH 9.0], 1% bentonite, and 1% Celite), as described previously (32, 35). Each plant was mechanically inoculated with 8 μl of the transcript-inoculation buffer mixture and maintained under dark conditions at 25°C overnight. The plants were then transferred to the diurnal growth chamber conditions for the duration of the infection cycle.

### RNA isolation, cDNA synthesis, and semiquantitative RT-PCRs.

Total RNA was isolated from *Brachypodium*, proso millet, and St. Augustinegrass samples as described previously using Direct-zol RNA miniprep kits (Zymo Research) with TRI reagent (Ambion) (28, 35). RNA quality was assessed using NanoDrop absorption values and by electrophoresis on 1% agarose gels, followed by ethidium bromide staining. Approximately 1 μg of total RNA from each sample was used as the template for cDNA preparation using SuperScript III reverse transcriptase (Invitrogen). Total cDNA was synthesized using oligo(dT) primers or random DNA hexamers where indicated. Semiquantitative RT-PCRs were performed using *Taq* DNA polymerase (NEB) with standard reaction conditions and appropriate primers (see Table S2 in the supplemental material). The PCRs were performed under the following conditions: 95°C for 30 s, 17 to 20 cycles of 95°C denaturation for 30 s, 52°C annealing for 30 s, and 68°C extension for 90 s, and a final extension at 68°C for 5 min. The PCR cycles were optimized to visually distinguish relative abundances of amplified cDNAs within a 1% agarose gel.

### Cloning and sequencing of the PMV poly(A) 3′ termini.

The initial *Brachypodium* samples analyzed (Fig. 2A) were collected at ∼10 to 14 days postinoculation (dpi), as part of a previous study (32). For subsequent experiments (Fig. 2B), noninoculated tissues of three symptomatic *Brachypodium* plants were pooled at 10, 21, and 42 dpi and used for total RNA isolation and cDNA preparation. For sequencing, RT steps were performed with a modified oligo(dT) primer for cDNA synthesis containing an overhanging adapter sequence for subsequence PCR amplification (OligodT-Adapter) (Table S2). PCRs were performed as described above with a forward primer corresponding to the 3′ end of the PMV CP ORF [PMV-poly(A)-F] (Table S2) and a reverse primer corresponding to the oligo(dT) overhanging adapter sequence (OligodT-Adapter-R) (Table S2). PCR products were gel purified and ligated into linearized pGEM-T vectors (Promega). Sequencing of the PMV 3′-end amplicon inserts was performed at the Texas A&M University Gene Technologies Laboratory using an ABI 3100 automated sequencer (Applied Biosystems).

### Identification of PMV and SPMV sequences in the SRA database.

The blastn suite at the NCBI SRA was used to mine the transcriptome data sets for mock-, PMV-, and PMV-SPMV-infected *Brachypodium* in the SRA (accession no. SRX746906, SRX747740, and SRX747746, respectively). The full-length reference genomes of PMV (NC_002598.1) and SPMV (NC_003847.1) were used as search queries. Search parameters were optimized for blastn, megablast, and discontiguous megablast to identify the maximum number of reads for each query and data set (Table S1). Searches were also performed on the *Brachypodium* data sets using the tblastn suite with the capsid protein amino acid sequences of PMV (NP_068346.1) and SPMV (NP_620827.1).

### Contig assemblies from short reads and sequence analyses.

A search of the PMV-infected *Brachypodium* mRNA-enriched transcriptome data set (accession no. SRX747740) yielded >20,000 reads with sequence similarity to the PMV genome (NC_002598.1). These reads were assembled into two preliminary contigs using the CodonCode Aligner desktop sequence assembly software (www.codoncode.com/aligner). Contig1 had a length of 2,384 bases and was assembled from 10,847 reads, while contig2 had a length of 1,905 bases and was assembled from 9,153 reads. Contig1 was used as query for megablast in NCBI. Hits included the 3′ end of the PMV genome (plus/plus orientation), the 3′ ends of *Thin paspalum asymptomatic virus* (TPAV) isolates, and the 3′ ends of the PMV chimeric satellite RNAs. Contig1 had >99% sequence identity with the PMV reference genome, with one mutation at position 3752 (U3752C). Contig2 was used as query for megablast in NCBI. Three hits were identified, including the 5′ end of the PMV reference genome (plus/minus orientation) and two TPAV isolates. Two additional mutations were identified within contig2 (G848U and U1799G). The terminal 9 bases at the 5′ end of the PMV reference genome were not identified in contig2.

A search of the PMV-SPMV-infected *Brachypodium* mRNA-enriched transcriptome data set (accession no. SRX747746) yielded 19,891 reads with sequence similarity to the PMV genome (Table S1). These reads were assembled into two contigs using CodonCode Aligner. Contig1 was assembled from 19,862 reads and was 4,669 bases long, while contig 2 was assembled from only 7 reads and was 226 bases long. There were 22 additional reads that were not assembled into either contig. Contig1 corresponded to the entire PMV genome (plus/minus orientation) with the same three mutations described above. Contig2 contained four partially overlapping fragments at the 5′ end of the PMV genome from positions 773 to 900, encompassing the region with the G848U mutation.

A search of the PMV-SPMV-infected *Brachypodium* poly(A)-selected transcriptome data set (accession no. SRX747746) was performed using the SPMV reference genome as the query (NC_003847.1). This search yielded 763 reads (Table S1), which were assembled into a single contig using the Web-based CAP3 sequence assembly program with standard parameters (doua.prabi.fr/software/cap3). This contig encompassed the majority of the SPMV genome, with multiple substitution and deletion mutations throughout. The contig had 16 nonviral bases extending from the 5′ end of the genome and 5 bases missing from the 3′ end.

### PMV and SPMV virion purification.

PMV and SPMV virions were purified from transcript-inoculated proso millet tissues as described previously, with minor modifications (31, 84). Infected shoot tissues were harvested at 14 dpi and stored at –80°C for approximately 4 weeks prior to virus purification. The infected plant tissues (15 to 20 g) were homogenized in 0.1 M potassium phosphate buffer, pH 7.4 (PPB), with 1.5% 2-mercaptoethanol, and the crude extracts were clarified by cheesecloth filtration and low-speed centrifugation (10,000 × *g*) for 10 min. Virions were concentrated by high-speed centrifugation (45,000 rpm, Ti60 rotor, 4°C) on a 20% sucrose cushion for 2 h. Virus pellets were resuspended in 50 mM PPB and subjected to a second low-speed centrifugation (10,000 × *g*) for 10 min. The clarified supernatant was loaded onto 10% to 40% sucrose density gradients in 50 mM PPB, and the virions were separated by high-speed centrifugation (35,000 rpm, SW41 rotor, 4°C) for 2 h. Following the density gradient separation, the light scattering bands for PMV and SPMV were removed and concentrated by a third high-speed centrifugation step (45,000 rpm, Ti60 rotor, 4°C) for 3 h in 50 mM PPB. The pelleted purified virions were resuspended in 1 ml of 50 mM PPB and stored at 4°C for further RT-PCR analysis.
