# Supplementary material for: Panicum Mosaic Virus and Its Satellites Acquire RNA Modifications Associated with Host-Mediated Antiviral Degradation
Source: mBio. 2019 Aug 27;10(4):e01900-19. doi: 10.1128/mBio.01900-19 (PMC6712398; doi:10.1128/mBio.01900-19)
Supplement: TABLE S1 [file mBio.01900-19-st001.docx]

| **Query Sequence** | **Host Organism** | **SRA Accession** | **Total Dataset Reads** | **Experimental Description** | **BLAST Stringency** | **# Reads** |
| --- | --- | --- | --- | --- | --- | --- |
|  |  |  |  |  |  |  |
| PMV genome (NC_002598.1) | *Brachypodium distachyon* | SRX746906 | 20,252,166 | **Mock**-infected, lab grown, polyA selection | Megablast | 5 |
| PMV genome (NC_002598.1) | *Brachypodium distachyon* | SRX747740 | 18,988,712 | **PMV**-infected, lab grown, polyA selection | Megablast | 20000 |
| PMV genome (NC_002598.1) | *Brachypodium distachyon* | SRX747746 | 20,570,446 | **PMV+SPMV**-infected, lab grown, polyA selection | Megablast | 19891 |
|  |  |  |  |  |  |  |
| PMV genome (NC_002598.1) | *Brachypodium distachyon* | SRX746906 | 20,252,166 | **Mock**-infected, lab grown, polyA selection | Discontiguous megablast | 5 |
| PMV genome (NC_002598.1) | *Brachypodium distachyon* | SRX747740 | 18,988,712 | **PMV**-infected, lab grown, polyA selection | Discontiguous megablast | 20000 |
| PMV genome (NC_002598.1) | *Brachypodium distachyon* | SRX747746 | 20,570,446 | **PMV+SPMV**-infected, lab grown, polyA selection | Discontiguous megablast | 19917 |
|  |  |  |  |  |  |  |
| PMV genome (NC_002598.1) | *Brachypodium distachyon* | SRX746906 | 20,252,166 | **Mock**-infected, lab grown, polyA selection | blastn | 5 |
| PMV genome (NC_002598.1) | *Brachypodium distachyon* | SRX747740 | 18,988,712 | **PMV**-infected, lab grown, polyA selection | blastn | 20000 |
| PMV genome (NC_002598.1) | *Brachypodium distachyon* | SRX747746 | 20,570,446 | **PMV+SPMV**-infected, lab grown, polyA selection | blastn | 19928 |
|  |  |  |  |  |  |  |
|  |  |  |  |  |  |  |
| SPMV genome (NC_003847.1) | *Brachypodium distachyon* | SRX746906 | 20,252,166 | **Mock**-infected, lab grown, polyA selection | Megablast | 0 |
| SPMV genome (NC_003847.1) | *Brachypodium distachyon* | SRX747740 | 18,988,712 | **PMV**-infected, lab grown, polyA selection | Megablast | 0 |
| SPMV genome (NC_003847.1) | *Brachypodium distachyon* | SRX747746 | 20,570,446 | **PMV+SPMV**-infected, lab grown, polyA selection | Megablast | 755 |
|  |  |  |  |  |  |  |
| SPMV genome (NC_003847.1) | *Brachypodium distachyon* | SRX746906 | 20,252,166 | **Mock**-infected, lab grown, polyA selection | Discontiguous megablast | 0 |
| SPMV genome (NC_003847.1) | *Brachypodium distachyon* | SRX747740 | 18,988,712 | **PMV**-infected, lab grown, polyA selection | Discontiguous megablast | 0 |
| SPMV genome (NC_003847.1) | *Brachypodium distachyon* | SRX747746 | 20,570,446 | **PMV+SPMV**-infected, lab grown, polyA selection | Discontiguous megablast | 759 |
|  |  |  |  |  |  |  |
| SPMV genome (NC_003847.1) | *Brachypodium distachyon* | SRX746906 | 20,252,166 | **Mock**-infected, lab grown, polyA selection | blastn | 0 |
| SPMV genome (NC_003847.1) | *Brachypodium distachyon* | SRX747740 | 18,988,712 | **PMV**-infected, lab grown, polyA selection | blastn | 0 |
| SPMV genome (NC_003847.1) | *Brachypodium distachyon* | SRX747746 | 20,570,446 | **PMV+SPMV**-infected, lab grown, polyA selection | blastn | 763 |
|  |  |  |  |  |  |  |
|  |  |  |  |  |  |  |
| PMV CP (NP_068346.1) | Brachypodium distachyon | SRX746906 | 20,252,166 | **Mock**-infected, lab grown, polyA selection | tblastn | 2 |
| PMV CP (NP_068346.1) | Brachypodium distachyon | SRX747740 | 18,988,712 | **PMV**-infected, lab grown, polyA selection | tblastn | 3922 |
| PMV CP (NP_068346.1) | Brachypodium distachyon | SRX747746 | 20,570,446 | **PMV+SPMV**-infected, lab grown, polyA selection | tblastn | 3716 |
|  |  |  |  |  |  |  |
| SPCP (NP_620827.1) | Brachypodium distachyon | SRX746906 | 20,252,166 | **Mock**-infected, lab grown, polyA selection | tblastn | 0 |
| SPCP (NP_620827.1) | Brachypodium distachyon | SRX747740 | 18,988,712 | **PMV**-infected, lab grown, polyA selection | tblastn | 0 |
| SPCP (NP_620827.1) | Brachypodium distachyon | SRX747746 | 20,570,446 | **PMV+SPMV**-infected, lab grown, polyA selection | tblastn | 497 |
|  |  |  |  |  |  |  |

**Table S1.** Summary of Sequence Read Archive (SRA) mining search parameters and results.
