# Supplementary figures and images for: Panicum Mosaic Virus and Its Satellites Acquire RNA Modifications Associated with Host-Mediated Antiviral Degradation
Source: mBio. 2019 Aug 27;10(4):e01900-19. doi: 10.1128/mBio.01900-19 (PMC6712398; doi:10.1128/mBio.01900-19)

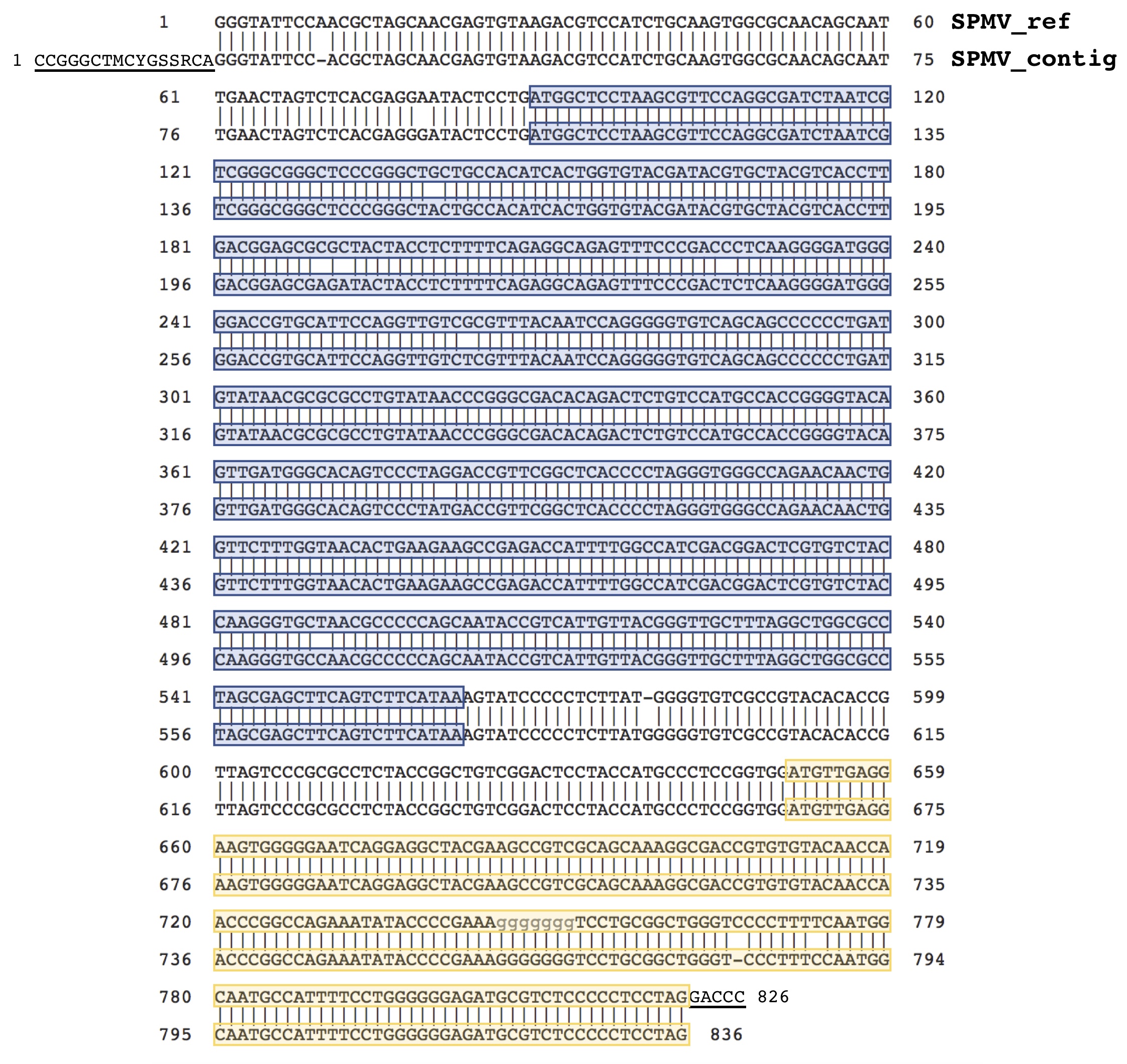

Supplement: FIG S1 [file mBio.01900-19-sf001.jpg]

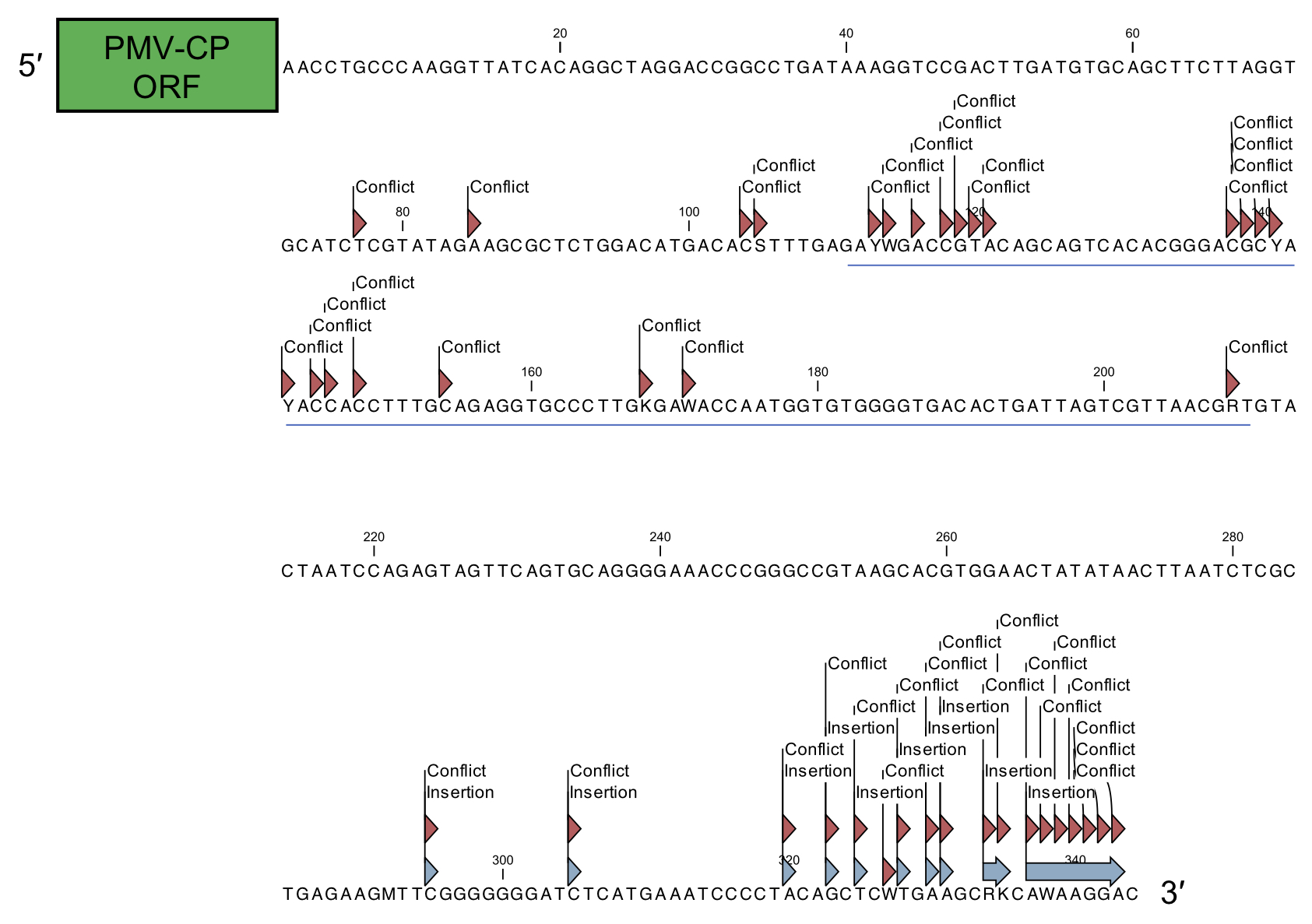

Supplement: FIG S2 [file mBio.01900-19-sf002.jpg]
