# Supplementary material for: Panicum Mosaic Virus and Its Satellites Acquire RNA Modifications Associated with Host-Mediated Antiviral Degradation
Source: mBio. 2019 Aug 27;10(4):e01900-19. doi: 10.1128/mBio.01900-19 (PMC6712398; doi:10.1128/mBio.01900-19)
Supplement: TABLE S2 [file mBio.01900-19-st002.docx]

| **Primer** | **Use in this study** | **Sequence (5' to 3')** |
| --- | --- | --- |
| PMV-p48-F | RT-PCR detection | ATGGAGTCGCTCATACAGCACA |
| PMV-p48-R | RT-PCR detection | CTACTTGGCAAACCTCAGACCC |
| PMV-CP-F | RT-PCR detection | ATGAATCGCAATGGAGCTAC |
| PMV-CP-R | RT-PCR detection | TTATGCGCTAACCCCACTGA |
| PMV-p6.6-F | RT-PCR detection | ATGGCGACCGGCAAGTGCTA |
| PMV-p6.6-R | RT-PCR detection | TCATTTCTGGATCTCAATGT |
| PMV-p8-F | RT-PCR detection | ATGTCTACTGTTGAGACTCC |
| PMV-p8-R | RT-PCR detection | CTAGAAATTGAAGTTGAAAG |
| SPMV-87-F | RT-PCR detection | ATGGCTCCTAAGCGTTCCA |
| SPMV-297-R | RT-PCR detection | ATACAGGCGCGCGTTATACATC |
| SPMV-297-F | RT-PCR detection | GATGTATAACGCGCGCCTGTAT |
| SPMV-541-R | RT-PCR detection | TTATGAAGACTGAAGCTCGC |
| SPMV-CP-F | RT-PCR detection | ATGGCTCCTAAGCGTTCC |
| SPMV-CP-R | RT-PCR detection | TTATGAAGACTGAAGCTCGC |
| SatC-F | RT-PCR detection | CTCAGGGAGGGGGTGTTGTC |
| SatC-R | RT-PCR detection | CCCCAATCTTCTCAGCGAGATT |
| PMV-polyA-F | PolyA Cloning | ATCAGTGGGGTTAGCGCATAA |
| OligodT-Adapter | PolyA Cloning | AAGCAGTGGTATCAACGCAGAGTACTTTTTTTTTTTTTTTTTTTTTTTTTTTTTT |
| OligodT-Adapter-R | PolyA Cloning | AAGCAGTGGTATCAACGCAGAGTAC |

**Table S2.** Sequences of oligonucleotides used in the current study. Forward and reverse primers for each set are indicated with “-F” and “-R” trailing the primer name, respectively.
